# Long-term findings of rectal endoscopy and rectal bleeding after moderately hypofractionated, intensity-modulated radiotherapy for prostate cancer

**DOI:** 10.1038/s41598-023-43202-x

**Published:** 2023-12-13

**Authors:** Bong Kyung Bae, Ji Eun Kim, Hongryull Pyo, Sung Noh Hong, Won Park

**Affiliations:** 1 Department of Radiation Oncology, Samsung Medical Center, Sungkyunkwan University School of Medicine, Seoul, Republic of Korea; 2 Department of Medicine, Samsung Medical Center, Sungkyunkwan University School of Medicine, Seoul, Republic of Korea; 3 Department of Radiation Oncology, Kyungpook National University Chilgok Hospital, Daegu, Republic of Korea

**Keywords:** Radiotherapy, Prostate cancer, Colonoscopy

## Abstract

To present rectal endoscopic findings and toxicity after definitive moderately hypofractionated, intensity-modulated radiotherapy (IMRT) for prostate cancer. We retrospectively reviewed patients who underwent IMRT for prostate cancer and underwent post-radiotherapy endoscopies between 2008 and 2018. Endoscopic findings were reviewed and graded using Vienna Rectoscopy Score (VRS). We have analyzed the association between endoscopic findings and rectal bleeding, and investigated risk factors for rectal bleeding. Total 162 patients met the inclusion criteria of this study. There was a trend of VRS worsening during the initial 3 years after radiotherapy followed by recovery. Rectal bleeding was highest at 1 year after radiotherapy and improved thereafter. The 5-year cumulative incidence of grade ≥ 2 rectal bleeding was 14.8%. In the multivariable Cox regression analysis, cardiovascular disease (hazard ratio [HR] 2.732, *P* = 0.037), rectal wall V_65_ (HR 1.158, *P* = 0.027), and VRS ≥ 3 in first post-radiotherapy endoscopy (HR 2.573, *P* = 0.031) were significant risk factors for rectal bleeding. After IMRT for prostate cancer, VRS and rectal bleeding worsened over 1–3 years after radiotherapy and recovered. Cardiovascular disease, rectal wall V_65_, and VRS ≥ 3 in first post-radiotherapy endoscopy were significant risk factors for rectal bleeding.

## Introduction

Prostate cancer was the second most common cancer and the fifth leading cause of cancer-related deaths in men in 2020^1^. Radiotherapy is a standard treatment option for localized and locally advanced prostate cancer^2–4^. As prostate cancer has low α/β ratio, larger dose per fraction for prostate cancer is potentially associated with increased therapeutic effects^5^. Multiple randomized trials comparing hypofractionation with conventional fractionation for prostate cancer have proven the non-inferiority of hypofractionation in clinical outcomes^6–8^.

Rectal bleeding is a major late side effect of radiotherapy for prostate cancer^9^. Most incidences of bleeding are temporary and self-limiting. However, some patients experience repeated episodes of bleeding that require interventions such as sucralfate enemas, endoscopic argon plasma coagulation (APC), blood transfusion, or even hospitalization^10^. Endoscopic examination provides objective findings of post-radiotherapy changes in the rectum^11^. Several studies have reported endoscopic changes after radiotherapy^11–13^. However, most studies have focused on the early changes or endoscopic findings at the time of toxic events. Long-term endoscopic changes, especially after hypofractionated radiotherapy, have not been reported.

Therefore, in this study, we evaluated the long-term endoscopic findings after definitive moderately hypofractionated intensity-modulated radiotherapy (IMRT) for prostate cancer to better understand changes in the rectal mucosa after radiotherapy. We also evaluated the correlation between endoscopic findings and rectal bleeding, and investigated the risk factors of rectal bleeding.

## Materials and methods

### Study design

With approval from the Institutional Review Board of the Samsung Medical Center (SMC IRB 2022-06-079), we retrospectively reviewed the medical records of patients who underwent definitive radiotherapy for localized or locoregional prostate cancer between January 2008 and December 2018 at Samsung Medical Center. Patients who underwent moderately hypofractionated IMRT with 70 Gy in 28 fractions and at least two post-radiotherapy endoscopies were included in this study. Patients who underwent single post-radiotherapy endoscopic evaluation was excluded from current study, as single endoscopic assessment may not adequately reflect rectal mucosal changes over time. Patients who received dose fractionation regimens other than 70 Gy in 28 fractions or those who lacked post-radiotherapy endoscopies were excluded.

### Radiotherapy

All patients underwent planning computed tomography (CT) simulations in the supine position with a 2.5 mm slice thickness. Rectal catheter with inflatable balloon was used for prostate immobilization. The balloon was inflated with 60 cc of air for simulation and daily treatment. Planning magnetic resonance imaging (MRI) was performed immediately after planning CT for target contouring assistance with the same setup position and immobilization devices. The CT and MRI images were automatically matched, and the images were checked in all directions and modified, if necessary, by a primary physician.

Target volume delineation and dose prescription principles were as follows. Patients with localized prostate cancer were stratified into one of three risk groups by the D’Amico risk classification^14^. For patients with low- and intermediate-risk prostate cancer, the prostate gland was contoured as the clinical target volume (CTV)-prostate. For patients with localized high-risk and locoregional prostate cancer, two CTVs were contoured. The prostate gland and the involved seminal vesicle were contoured as the CTV-prostate. The elective pelvic lymph node volume up to the sacral promontory was contoured as CTV-pelvis, as recommended by Lawton et al.^15^. If a metastatic lymph node was present, it was contoured as the GTV-lymph node (LN). The planning target volume (PTV) expansion from CTVs was a 5 mm expansion from the CTV-prostate and the GTV-LN for the PTV-prostate and the PTV-LN, and a 7 mm expansion from the CTV-pelvis for the PTV-pelvis. With consideration of dose constraints of normal organs, prescribed doses were as follows: (1) PTV-prostate: 70 Gy in 28 fractions, 2.5 Gy per fraction; (2) PTV-LN: 61.6 Gy to 70 Gy in 28 fractions, 2.2 to 2.5 Gy per fraction; (3) PTV-pelvis: 50.4 Gy in 28 fractions, 1.8 Gy per fraction. The rectal wall was contoured above and below 1 cm from the PTV-prostate. The dose constraints applied to the rectal wall were D_max_ < 74 Gy, V_70_ ≤ 7%, V_65_ ≤ 10%, V_60_ ≤ 15%, V_50_ ≤ 20%, and V_25_ ≤ 50%.

Image-guided radiotherapy was performed with daily pretreatment cone-beam computed tomography to reduce setup uncertainties. Rectal balloon was always inserted at the same depth with same inflated volume every treatment. The balloon was used as a surrogate for the location of target volumes. Fiducial markers or rectal spacers were not used for the patients in the study period.

### Assessments

Baseline clinical and treatment characteristics including age, Eastern Cooperative Oncology Group Performance Status, comorbidities, use of antithrombotic medication, initial prostate-specific antigen (PSA) level, stage of prostate cancer, Gleason score, radiotherapy volume, treatment modality, use of androgen deprivation therapy, and dose-volume histogram (DVH) parameters of the rectal wall were collected.

**Figure 1:**
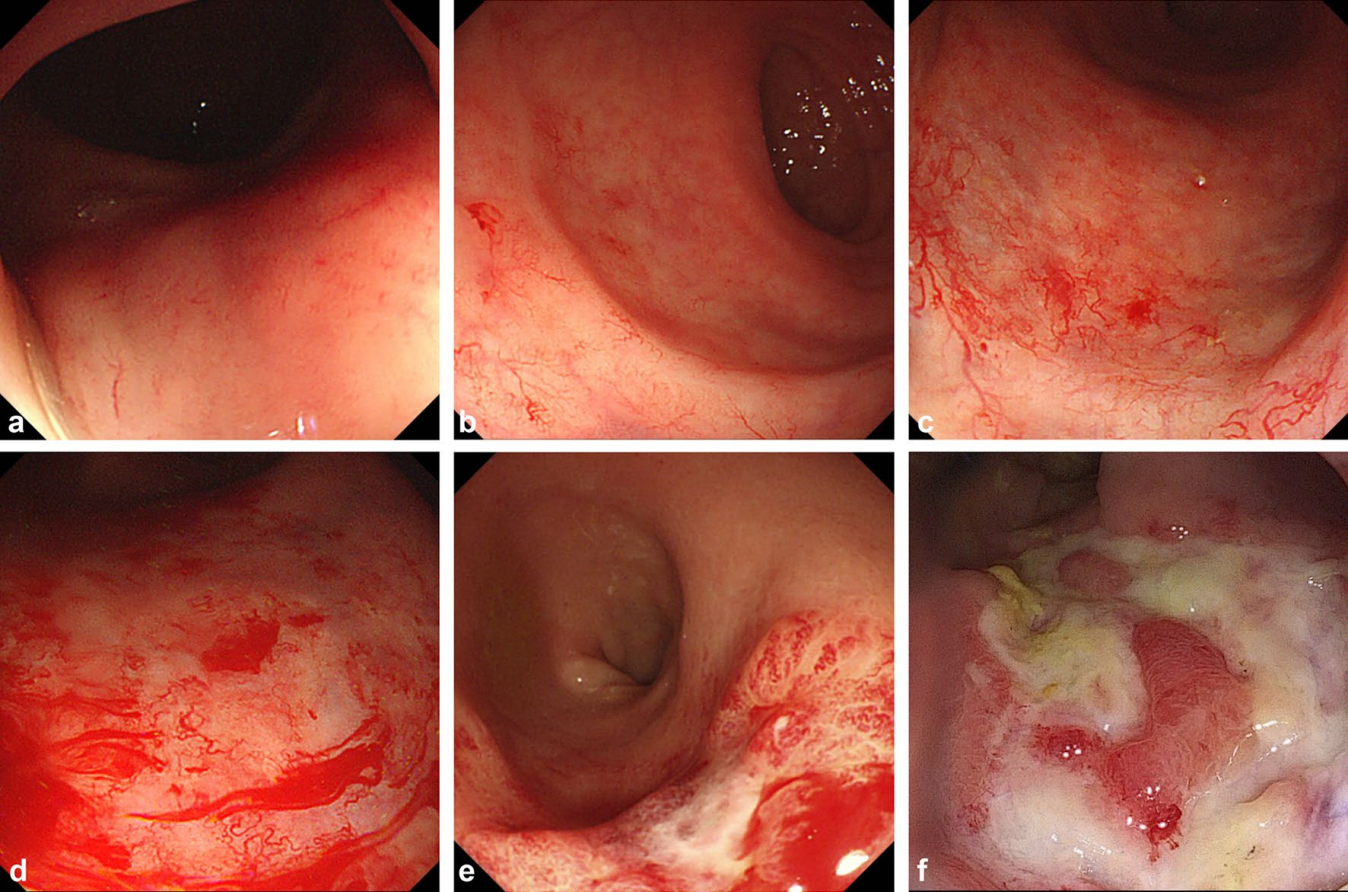
Endoscopic findings of respective VRS: (**a**) VRS 0 with grade 1 mucosal congestion, (**b**) VRS 1 with grade 1 telangiectasia, (**c**) VRS 2 with grade 2 telangiectasia, (**d**) VRS 3 with grade 3 telangiectasia, (**e**) VRS 4 with grade 2 ulceration, (**f**) VRS 5 with grade 3 ulceration. VRS, Vienna Rectoscopy Score.

Patients were recommended to visit the follow-up clinic every 3 months for the first 2 years, every 6 months for the next 3 years, and annually thereafter. During follow-ups, the patients were requested to undergo rectosigmoidoscopy or colonoscopy annually for 5 years after treatment. Endoscopic findings of the rectal mucosa were reviewed and graded by an experienced gastroenterology endoscopy specialist (J.E.K.) using the Vienna Rectoscopy Score (VRS) suggested by Wachter et al.^16^. According to the VRS, five different endoscopic components of mucosal damage (mucosal congestion, telangiectasia, ulceration, stricture, and necrosis) are graded from grade 0 to 3. Based on the grades of the individual parameters, VRS is derived as a six-scaled score, from 0 to 5. Examples of the VRS are shown in Fig. 1. Symptomatic rectal bleeding was graded according to the Common Terminology Criteria for Adverse Events, version 5.0: grade 1, mild symptoms with no intervention is indicated; grade 2, moderate symptoms with interventions such as APC is indicated; grade 3, transfusion, invasive intervention, or hospitalization is indicated; grade 4, life-threatening incidences; grade 5, death. Changes in endoscopic findings and severity of rectal bleeding in individual patients were collected and analyzed on the basis of the time from the end of radiotherapy. Endoscopy performed in the first year after radiotherapy was regarded as the first post-radiotherapy endoscopy, and endoscopic findings were used in the investigation of the risk factors for rectal bleeding.

### Statistical analyses

Correlation analysis with the Chi-square test and the Fisher’s exact test was performed to determine the association between endoscopic findings and rectal bleeding each year. The cumulative incidence of rectal bleeding was calculated and plotted using the Kaplan–Meier method. Cox regression analysis and binary logistic regression analysis were performed to determine factors associated with grade ≥ 2 rectal bleeding and worst VRS ≥ 3. Factors with *P*-value < 0.10 in the univariable analysis were considered as potential candidates for multivariable analysis. Out of potential candidates, variables with a variance inflation factor < 10.0 entered the multivariable analysis. The final multivariable model was determined using a backward variable selection method. Statistical significance was set at *P*-value < 0.05. Statistical analyses were performed using the IBM SPSS Statistics software (version 27.0; IBM, Inc., Armonk, NY, USA).

### Ethics approval

The institutional review board of the Samsung Medical Center approved the study. Informed consent was waived by the review board due to the retrospective nature of the study. All procedures performed involving human participants were in accordance with the Declaration of Helsinki as revised in 2013.

## Results

### Patient and treatment characteristics

A total of 162 patients met the inclusion criteria of this study. The flowchart of patient selection process is illustrated in Supplementary Fig. 1. The median follow-up time was 4.8 years (range, 3.1−10.9 years). The median age of the patients was 73 years (interquartile range, 67−77 years) and the mean initial PSA was 26.6 ng/mL. A total of 53 patients (32.7%) were under antithrombotic medication at the time of radiotherapy with antiplatelet drugs (48 patients, 29.6%), anticoagulant drugs (3 patients, 1.9%), or both (2 patients, 1.2%). The clinical T stage was T1 in 5 (3.1%), T2 in 64 (39.5%), T3 in 85 (52.5%), and T4 in 8 patients (4.9%). The clinical N stage was N0 in 135 (83.3%) and N1 in 27 patients (16.7%). The risk groups of patients were low-risk in 13 (8.0%), intermediate-risk in 47 (29.0%), high-risk in 75 (46.3%), and locally advanced disease in 27 patients (16.7%). The radiotherapy target volume was prostate gland ± seminal vesicle in 80 patients (49.4%) and whole pelvis in 82 patients (50.6%). Androgen deprivation therapy was administered to 84 patients (51.8%). Baseline patient and treatment characteristics are summarized in Table 1.

**Table 1:** Baseline patient and treatment characteristics.

Variable	Category	Total
Age (median, IQR)		73 (67–77)
ECOG PS		
	0	138 (85.2%)
	1	24 (14.8%)
Comorbidity		
	DM	30 (18.5%)
	HTN	82 (50.6%)
	Cerebrovascular disease	11 (6.8%)
	Cardiovascular disease	23 (14.2%)
Antithrombotic medication		
	No	109 (67.3%)
	Antiplatelet	48 (29.6%)
	Anticoagulant	3 (1.9%)
	Both	2 (1.2%)
Initial PSA (ng/mL, Mean ± SD)		26.6 ± 44.6
cT stage		
	1	5 (3.1%)
	2	64 (39.5%)
	3	85 (52.5%)
	4	8 (4.9%)
cN stage		
	0	135 (83.3%)
	1	27 (16.7%)
Gleasons score		
	6	36 (22.2%)
	7	58 (35.8%)
	8	44 (27.2%)
	9	23 (14.2%)
	10	1 (0.6%)
NCCN risk group		
	Low	13 (8.0%)
	Intermediate	47 (29.0%)
	High	75 (46.3%)
	Locally advanced	27 (16.7%)
Radiotherapy volume		
	Prostate ± seminal vesicle	80 (49.4%)
	Whole pelvis	82 (50.6%)
Radiotherapy modality		
	Photon	158 (97.5%)
	Proton	4 (2.5%)
ADT		
	No	78 (48.2%)
	Yes	84 (51.8%)
DVH parameters (Mean ± SD)		
	CTV-prostate (cc)	44.3 ± 19.1
	Rectal wall V_25_ (cc)	20.8 ± 15.7
	Rectal wall V_50_ (cc)	7.7 ± 5.1
	Rectal wall V_60_ (cc)	4.9 ± 2.8
	Rectal wall V_65_ (cc)	3.5 ± 1.8
	Rectal wall V_70_ (cc)	1.4 ± 0.9
	Rectal wall V_25_ (%)	43.3 ± 12.8
	Rectal wall V_50_ (%)	17.3 ± 5.9
	Rectal wall V_60_ (%)	11.5 ± 4.1
	Rectal wall V_65_ (%)	8.6 ± 3.6
	Rectal wall V_70_ (%)	3.5 ± 2.4
	Rectal wall D_max_ (Gy)	72.0 ± 0.8

ECOG PS, Eastern Cooperative Oncology Group Performance Status; DM, diabetes; HTN, hypertension; PSA, prostate specific antigen; NCCN, National Comprehensive Cancer Network; ADT, androgen deprivation therapy; DVH, dose volume histogram; CTV, clinical target volume.

### Endoscopic findings

Annual post-radiotherapy endoscopy was performed a median of four-times per patient (range, 2−5). The following number of patients underwent endoscopy each year: 149 in the first year (92.0%), 153 in the second year (94.4%), 129 in the third year (79.6%), 87 in the fourth year (53.7%), and 53 in the fifth year (32.7%).

**Figure 2:**
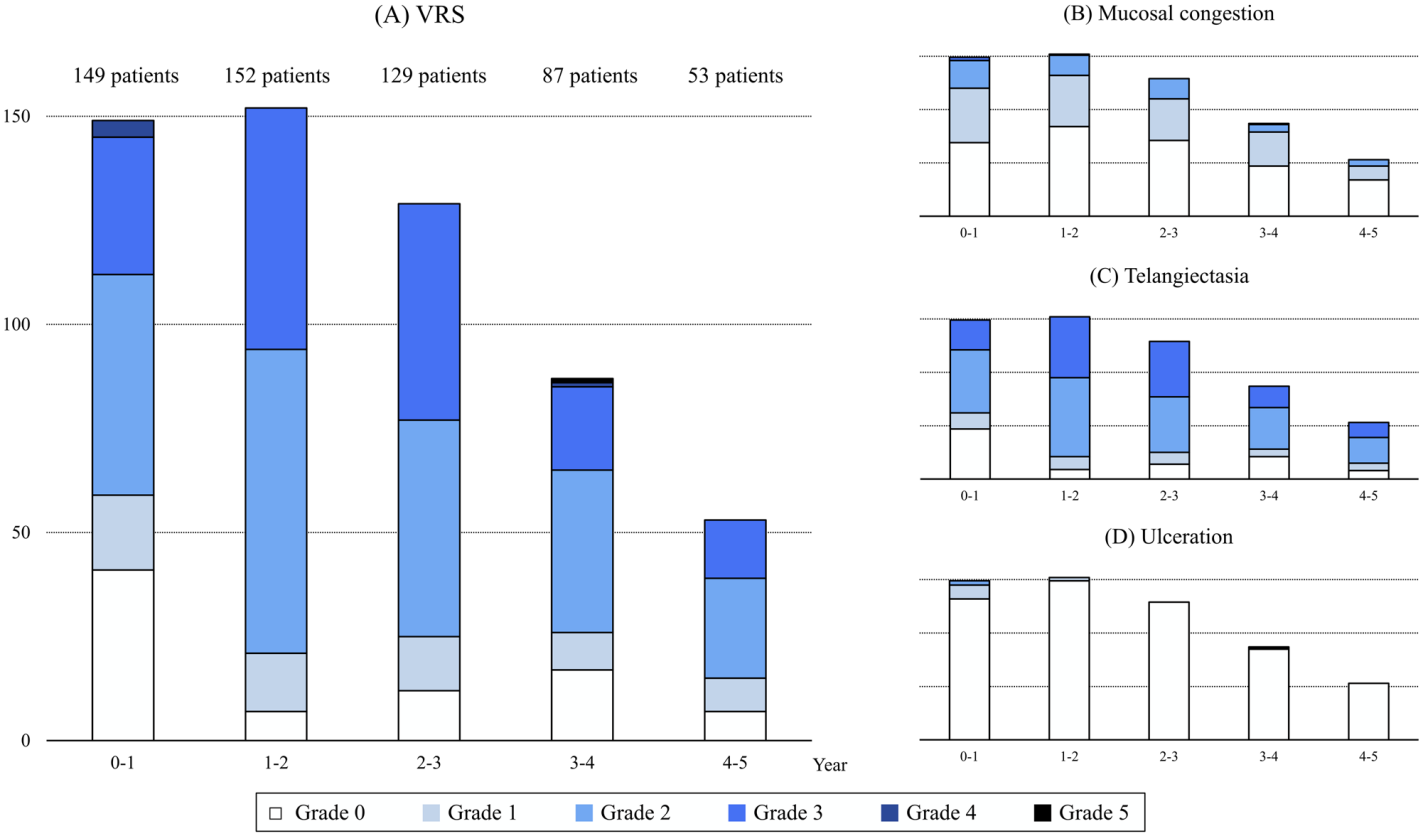
Changes in VRS and endoscopic findings after radiotherapy. Endoscopic mucosal changes worsened over the first 2−3 years after radiotherapy, and gradually recovered afterwards. (**a**) overall VRS, (**b**) mucosal congestion, (**c**) telangiectasia, and (**d**) ulceration. VRS, Vienna Rectoscopy Score.

There was a trend of worsening VRS during the initial 3 years after radiotherapy, which recovered afterwards. VRS ≥ 2 was observed in 60.4%, 86.2%, 80.6%, 70.1%, and 71.7% of the patients annually. VRS ≥ 3 was observed in 24.8%, 38.2%, 40.3%, 25.3%, and 26.4% of the patients annually. Out of the five individual parameters of mucosal damage, telangiectasia was the most prominent parameter. Grade ≥ 2 telangiectasia was observed in 58.4%, 86.2%, 80.6%, 67.8%, and 71.7% of the patients annually. Grade 3 telangiectasia was observed in 18.8%, 37.5%, 40.3%, 23.0%, and 26.4% of the patients annually. Strictures or necrosis were not observed. The details of the changes in each parameter are summarized in Fig. 2 and Supplementary Table 1.

### Rectal bleeding

**Figure 3:**
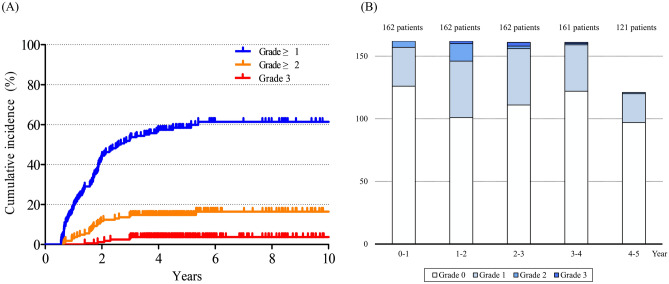
Rectal bleeding after radiotherapy. (**a**) Cumulative incidence of grade ≥ 1 (blue), grade ≥ 2 (orange), and grade 3 (red) rectal bleeding. (**b**) Changes in rectal bleeding after radiotherapy. Rectal bleeding was worst 1−2 years after radiotherapy, which recovered afterwards.

Rectal bleeding was observed in 95 patients (58.6%) after radiotherapy. Grade ≥ 2 rectal bleeding was observed in 25 patients (15.4%), and grade 3 rectal bleeding was observed in 6 patients (3.7%). The 5-year cumulative incidences of rectal bleeding were 58.4%, 14.8%, and 2.7% for Grade ≥ 1, Grade ≥ 2, and Grade 3. The proportion of patients with rectal bleeding was highest at 1−2 years after radiotherapy (grade 1 in 45 patients, grade 2 in 14 patients, and grade 3 in 2 patients), and the bleeding decreased afterwards. Although grade 2 patients required APC and grade 3 patients required transfusion or hospitalization, the events were well-managed without specific complications. The cumulative incidence of rectal bleeding and changes in rectal bleeding over time are summarized in Fig. 3.

### Correlation between endoscopic findings and rectal bleeding and risk factors for rectal bleeding

Endoscopic findings and rectal bleeding of individual patients were collected and analyzed on annual basis after radiotherapy to determine correlations between these factors. In the correlation analysis, there were statistically significant correlations between factors throughout the follow-up period (from 0−1 year to 3−4 years after radiotherapy, *P* < 0.001, and 4−5 years after radiotherapy, *P* = 0.049). The results are summarized in Table 2.

**Table 2:** Correlation analysis of endoscopic findings and rectal bleeding. Factors were compared on annual basis after radiotherapy.

		Late rectal bleeding at respective year		*P* value
		Grade 0	Grade ≥ 1	
VRS, 0 − 1 year	Score			< 0.001*
	0 − 2	95 (84.8%)	19 (51.4%)	
	3 − 5	17 (15.2%)	18 (48.6%)	
VRS, 1 − 2 year	Score			< 0.001*
	0 − 2	76 (80.9%)	20 (34.5%)	
	3 − 5	18 (19.1%)	38 (65.5%)	
VRS, 2 − 3 year	Score			< 0.001*
	0 − 2	60 (77.9%)	24 (46.2%)	
	3 − 5	17 (22.1%)	28 (53.8%)	
VRS, 3 − 4 year	Score			< 0.001*
	0 − 2	56 (86.2%)	7 (31.8%)	
	3 − 5	9 (13.8%)	15 (68.2%)	
VRS, 4 − 5 year	Score			0.049**
	0 − 2	29 (74.4%)	6 (42.9%)	
	3 − 5	10 (25.6%)	8 (57.1%)	

VRS, Vienna Rectoscopy Score.

*Chi-square test, **Fisher’s exact test.

Cox regression analysis was performed to identify risk factors associated with grade ≥ 2 rectal bleeding. In the univariable analysis, cardiovascular disease (hazard ratio [HR] 1.185, 95% confidence interval [CI] 1.111−6.377, *P* = 0.028) and rectal wall V_65_ (HR 1.171, 95% CI 1.032−1.329, *P* = 0.014) were significant risk factors for rectal bleeding. Multivariable analysis revealed that cardiovascular disease (HR 2.732, 95% CI 1.060−7.038, *P* = 0.037), rectal wall V_65_ (HR 1.158, 95% CI 1.017−1.318, *P* = 0.027), and VRS ≥ 3 in the first post-radiotherapy endoscopy (HR 2.573, 95% CI 1.091−6.069, *P* = 0.031) were significant risk factors for rectal bleeding. There was no clear cut-off value for rectal wall DVH parameters. The results are summarized in Table 3.

**Table 3:** Univariable and multivariable cox regression analysis of potential factors of grade 2 or higher rectal bleeding.

Variable	Univariable		Multivariable	
	HR (95% CI)	*P* value	HR (95% CI)	*P* value
Patient and treatment parameters				
Age (> 70)	1.456 (0.628–3.374)	0.381		
Diabetes (yes)	1.843 (0.769–4.416)	0.171		
Hypertension (yes)	0.893 (0.407–1.956)	0.777		
Cerebrovascular disease (yes)	1.185 (0.279–5.027)	0.818		
Cardiovascular disease (yes)	2.662 (1.111–6.377)	0.028	2.732 (1.060–7.038)	0.037
Antithrombotic medication (yes)	1.683 (0.764–3.708)	0.196		
RT volume (Whole pelvis)	0.765 (0.347–1.685)	0.506		
ADT (yes)	0.739 (0.335–1.627)	0.452		
DVH parameters				
CTV-prostate (cc, continuous)	1.004 (0.986–1.023)	0.657		
Rectal wall V_25_ (%, continuous)	1.004 (0.974–1.035)	0.803		
Rectal wall V_50_ (%, continuous)	1.032 (0.976–1.091)	0.269		
Rectal wall V_60_ (%, continuous)	1.110 (0.995–1.238)	0.061		
Rectal wall V_65_ (%, continuous)	1.171 (1.032–1.329)	0.014	1.158 (1.017–1.318)	0.027
Rectal wall V_70_ (%, continuous)	1.170 (0.987–1.386)	0.070		
Rectal wall D_max_ (Gy, continuous)	1.169 (0.734–1.863)	0.511		
Rectal wall V_25_ (> 50%)	0.997 (0.416–2.394)	0.995		
Rectal wall V_50_ (> 20%)	1.333 (0.588–3.021)	0.491		
Rectal wall V_60_ (> 15%)	2.173 (0.936–5.045)	0.071		
Rectal wall V_65_ (> 10%)	1.275 (0.579–2.811)	0.546		
Rectal wall V_70_ (> 7%)	2.995 (0.705–12.718)	0.137		
Rectal wall D_max_ (> 74 Gy)	1.579 (0.213–11.680)	0.655		
First post-RT endoscopic findings				
Congested mucosa (≥ G2)	1.595 (0.622–4.091)	0.332		
Telangiectasia (≥ G2)	2.050 (0.802–5.239)	0.134		
Ulceration (≥ G1)	0.752 (0.176–3.219)	0.701		
VRS (≥ 2)	2.399 (0.885–6.502)	0.086		
VRS (≥ 3)	2.326 (0.994–5.442)	0.052	2.573 (1.091–6.069)	0.031

RT, radiotherapy; ADT, androgen deprivation therapy; DVH, dose volume histogram; CTV, clinical target volume; VRS, Vienna Rectoscopy Score.

Binary logistic regression analysis was performed to find risk factors of worse endoscopic finding, VRS ≥ 3. Multiple DVH parameters of rectal wall were significantly associated with worse endoscopic findings in the univariable analysis: rectal wall V_25_ (OR 1.036, 95% CI 1.009–1.064, *P* = 0.009), rectal wall V_50_ (OR 1.106, 95% CI 1.036–1.182, *P* = 0.003), rectal wall V_60_ (OR 1.132, 95% CI 1.044–1.228, *P* = 0.003), rectal wall V_65_ (OR 1.154, 95% CI 1.052–1.266, *P* = 0.002), rectal wall V_70_ (OR 1.155, 95% CI 1.005–1.328, *P* = 0.042), respectively. Multivariable analysis showed that rectal wall V_25_ (OR 1.029, 95% CI 1.001–1.058, *P* = 0.039), rectal wall V_65_ (OR 1.134, 95% CI 1.030–1.248, *P* = 0.011) were significantly associated with worse endoscopic findings. The results of binary logistic regression analysis are summarized in Table 4.

**Table 4:** Univariable and multivariable binary logistic regression analysis of potential risk factors of VRS 3 or higher.

Variable	Univariable		Multivariable	
	OR (95% CI)	*P* value	OR (95% CI)	*P* value
Patient and treatment parameters				
Age (> 70)	1.259 (0.661–2.397)	0.484		
DM (yes)	0.915 (0.407–2.057)	0.829		
HTN (yes)	0.684 (0.362–1.294)	0.243		
Cerebrovascular disease (yes)	0.728 (0.212–2.493)	0.613		
Cardiovascular disease (yes)	1.497 (0.578–3.874)	0.406		
Antithrombotic medication (yes)	0.647 (0.331–1.263)	0.202		
RT volume (Whole pelvis)	1.283 (0.680–2.421)	0.441		
ADT (yes)	0.915 (0.485–1.725)	0.783		
DVH parameters				
High-risk CTV (cc, continuous)	0.944 (0.977–1.010)	0.446		
Rectal wall V_25_ (%, continuous)	1.036 (1.009–1.064)	0.009	1.029 (1.001–1.058)	0.039
Rectal wall V_50_ (%, continuous)	1.106 (1.036–1.182)	0.003		
Rectal wall V_60_ (%, continuous)	1.132 (1.044–1.228)	0.003		
Rectal wall V_65_ (%, continuous)	1.154 (1.052–1.266)	0.002	1.134 (1.030–1.248)	0.011
Rectal wall V_70_ (%, continuous)	1.155 (1.005–1.328)	0.042		
Rectal wall D_max_ (Gy, continuous)	1.252 (0.827–1.896)	0.288		
Rectal wall V_25_ (> 50%)	1.942 (0.928–4.063)	0.078		
Rectal wall V_50_ (> 20%)	1.481 (0.733–2.993)	0.274		
Rectal wall V_60_ (> 15%)	2.016 (0.839–4.844)	0.117		
Rectal wall V_65_ (> 10%)	2.000 (1.018–3.929)	0.044		
Rectal wall V_70_ (> 7%)	0.928 (0.151–5.715)	0.936		
Rectal wall D_max_ (> 74 Gy)	0.612 (0.084–4.462)	0.628		

VRS, Vienna Rectoscopy Score; RT, radiotherapy; ADT, androgen deprivation therapy; DVH, dose volume histogram; CTV, clinical target volume.

### Rectal bleeding and endoscopic findings of patients with initial VRS ≥ 3 over time

**Figure 4:**
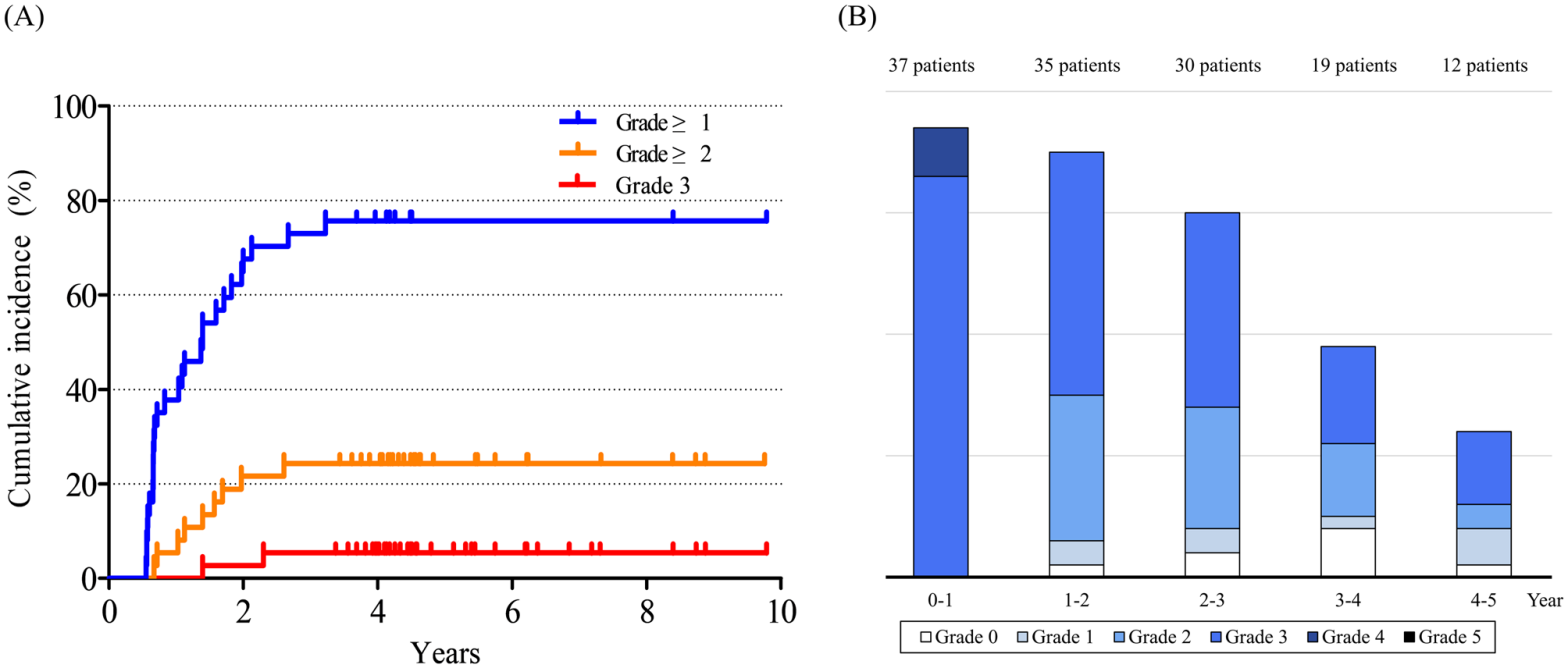
Rectal bleeding and endoscopic findings of patients with initial VRS ≥ 3. (**a**) Cumulative incidence of grade ≥ 1 (blue), grade ≥ 2 (orange), and grade 3 (red) rectal bleeding. (**b**) Changes in VRS over time. VRS, Vienna Rectoscopy Score.

Figure 4 shows the cumulative incidence of rectal bleeding and changes of VRS for 37 patients who presented VRS ≥ 3 in the first post-radiotherapy endoscopy (VRS 3 in 33, and VRS 4 in 4 patients). Compared to the study cohort, the group of patients showed significantly higher proportion of patients experiencing rectal bleeding (5-year cumulative incidence, 75.7%, 24.3%, and 5.4% for Grade ≥ 1, Grade ≥ 2, and Grade 3, respectively). Most severe toxic event observed was grade 3 in 2 patients (5.4%) who required blood transfusion due to low hemoglobin level from rectal bleeding. Grade 2 toxic events were observed in 7 patients (18.9%) who underwent APC. Other patients reported self-limited rectal bleeding not requiring active management (19 patients, 51.4%) or did not report any rectal bleeding (9 patients, 24.3%). Also, it is to note that the endoscopic findings improved over time without any significant deterioration.

## Discussion

In this study, we reviewed the endoscopic findings, rectal bleeding, and risk factors for rectal bleeding after moderately hypofractionated IMRT for prostate cancer. Endoscopic findings showed that rectal mucosal damage was prominent and worsened until 2−3 years after radiotherapy and recovered afterwards. The proportion of patients with rectal bleeding increased until 1−2 years after radiotherapy and decreased thereafter. We revealed that cardiovascular disease, rectal wall V_65_, and VRS ≥ 3 in the first post-radiotherapy endoscopy were risk factors of post-radiotherapy rectal bleeding.

The significance and usefulness of endoscopic rectal mucosal changes after radiotherapy have been studied in various clinical settings. Goldner et al. evaluated endoscopic changes after radiotherapy for prostate cancer^11^. After 70−74 Gy with 2 Gy per fraction, VRS ≥ 2 was observed in 46% and 33% of patients at 12 and 24 months, respectively. There was also a significant correlation between the maximal VRS and rectal side effects. Ohtani et al. reported long-term endoscopic changes and rectal bleeding after brachytherapy for prostate cancer^13^. The incidence of rectal bleeding was 24%, and there was a statistically significant correlation between VRS and rectal bleeding. Ippolito et al. performed proctoscopy 1 year after definitive or adjuvant radiotherapy for prostate cancer and investigated the role of early mucosal changes in predicting late rectal toxicity^12^. The 3-year cumulative incidence of grade ≥ 2 rectal toxicity was 24%, with a significant correlation with initial telangiectasia grade and VRS. They concluded that early proctoscopy could predict late rectal bleeding. There are also studies investigating the role of endoscopy after radiotherapy for cervical cancer and rectal cancer^17,18^.

In this study, VRS ≥ 3 was observed in 100 patients (61.7%) during follow-up, and 14 patients (26.4%) had a VRS 3 until 5 years after radiotherapy.

Compared to the above-mentioned studies, the mucosal damage of patients in our study seems to be relatively severe. There may be several reasons for this difference in mucosal damage. First, there were differences in the patient population. While our study is based on patients who underwent moderately hypofractionated IMRT for prostate cancer with 70 Gy in 28 fractions, previous studies are based on patients treated with conventional fractionated radiotherapy with 3 dimensional conformal radiotherapy (3D-CRT)^11^, both 3D-CRT and IMRT^12^, or brachytherapy^13^. Second, the number of patients with endoscopic evaluation decreased over time, with 149 patients (92.0%) and 152 patients (94.4%) in the first and second year, respectively, and 53 patients (32.7%) in the fifth year after radiotherapy. Also, out of 379 patients who underwent moderately hypofractionated IMRT for prostate cancer, 217 patients refused endoscopic evaluations. As endoscopic evaluation was recommended but not mandatory, there was a tendency of patients without specific complications to refuse further endoscopic evaluation. We speculate that mucosal damage in patients who did not underwent endoscopic evaluation would have improved significantly. Cumulative incidence of rectal bleeding supports the speculation. We have compared cumulative incidence of rectal bleeding between two cohorts: the study cohort (162 patients), and a cohort that includes all patients before the exclusion of those who did not undergo post-radiotherapy endoscopies (379 patients) (Supplementary Fig. 1). The cumulative incidence of rectal bleeding was higher for the study cohort (5-year incidence, Grade ≥ 1, 58.4% vs. 42.2%; Grade ≥ 2, 14.8% vs. 13.7%; Grade 3, 2.7% vs. 2.4%) (Supplementary Fig. 2). This suggests that a significant number of patients without any toxic events were excluded from current study due to the lack of endoscopic findings. This may have affected the high toxic events observed in the study cohort. However, we were unable to present clear evidence concerning this speculation because of lack of data.

Randomized trials and meta-analyses of hypofractionated radiotherapy for prostate cancer have reported outcomes similar to those of conventionally fractionated radiotherapy in terms of tumor control^6,19–22^. However, toxicity varies greatly between studies, with the reported incidence of grade ≥ 2 gastrointestinal (GI) toxicity ranging from 4 to 30%^22–25^. Direct comparison of toxic events with previously published studies is challenging, as the toxicity grading system, radiotherapy dose prescription, and patient population differ between studies. However, the incidence of rectal bleeding in this study seems to be similar to that reported in other studies (grade ≥ 2, 15.4%; grade 3, 3.7%; no grade 4 toxic events).

Mucosal damage and rectal bleeding after radiotherapy dynamically change over time. In this study, the proportion of patients with high VRS showed an increasing trend over the first 2−3 years after radiotherapy and decreased thereafter (Fig. 2a). The proportion of patients with rectal bleeding increased over the first 1−2 years after radiotherapy and decreased thereafter (Fig. 2b). Abdalla et al. reported that the peak incidence of GI toxicity was observed 2−3 years after radiotherapy for prostate cancer with 60−74 Gy^26^. Groen et al. reported that grade ≥ 2 GI toxicity increased in the first two years in patients of the FLAME trial^27,28^. The time of peak incidence differs between studies; however, it is to note that GI toxic events can occur for a prolonged period after the end of treatment.

Several studies have reported that anticoagulation therapy results in increased rectal bleeding after radiotherapy for prostate cancer. Choe et al. reported that patients taking warfarin or clopidogrel had a significantly increased risk of bleeding after radiotherapy for prostate cancer^29^. Takeda et al. reported that using anticoagulants or antiaggregants resulted in increased late rectal toxicity after radiotherapy for prostate cancer^30^. The findings were identical in a study by Kim et al., who reported an increased risk of grade ≥ 3 rectal bleeding for patients taking anticoagulants^4^. Unlike previous studies, as shown in Table 3, the use of antithrombotic medication was not a significant risk factor for rectal bleeding in the current study (HR 1.683, *P* = 0.196). However, cardiovascular disease was significantly associated with increased rectal bleeding (HR 2.732, *P* = 0.037). This might result from the differences in the types of antithrombotic medications administered. While most patients receiving antithrombotic medication were taking aspirin for the prevention of cardiovascular disease, patients with cardiovascular disease were taking various antithrombotic drugs in various combinations (from only aspirin to a combination of clopidogrel and new oral anticoagulants). The simple use of antithrombotic medications for the prevention of cardiovascular disease may not have to be considered as a risk factor for rectal bleeding after radiotherapy for prostate cancer; however, this will need further validation.

There are several strategies to reduce rectal toxicity in radiotherapy for prostate cancer. First, using rectal balloon results in prostate immobilization allowing smaller PTV margin and reduces rectal volume receiving high-dose radiation^31^. Second, fiducial marker can be placed for improving the accuracy of prostate targeting and reducing rectal toxicity^32^. Third, rectal spacer, which injects absorbable polyethylene glycol hydrogel spacer into the perirectal space, can physically move anterior rectal wall away from prostate, resulting in reduced rectal radiation dose and decreased radiotherapy related toxicities^33^. Fourth, if gastrointestinal toxic event occurs after radiotherapy, hyperbaric oxygen therapy might have potential to reduce the side effects^34,35^.

This study reports the long-term endoscopic findings after moderately hypofractionated IMRT for prostate cancer. We believe that this study is the largest study with long-term follow-ups with serial endoscopies. This study provides useful clinical information on actual rectal mucosal changes after radiotherapy for prostate cancer. However, this study had several limitations. First, the results could have been biased owing to the retrospective nature of the study and patients lost during follow-up. Median follow-up time of 4.8 years with shortest duration of 3.1 years seems to be sufficient for assessment of radiation-related late toxicity. We believe that further worsening of rectal toxicity after 3 years is unlikely. However, definitive confirmation is not possible due to the lack of data. Second, endoscopic findings were scored according to the interpretation of the images obtained during endoscopy. As the rectal mucosa is vulnerable to air inflation, which can cause bleeding, it is important to evaluate the mucosal status during the entrance of scope to avoid interference from the endoscopic procedure itself. However, it is difficult to identify if the image was taken during entrance or withdrawal of the scope, which could have led to over- or under-estimation of rectal mucosal findings. Third, comorbidities and medications of individual patients were assessed before treatment initiation, and changes during or after radiotherapy were not assessed. There may have been patients with new anticoagulant or antiplatelet medications, but those aspects were not considered in current study. Fourth, endoscopic evaluation of patients was recommended but not mandatory. The results could have been biased as quite a few patients were excluded from current study due to lack of endoscopic evaluation.

## Conclusion

After moderately hypofractionated IMRT for prostate cancer, the VRS worsened during the initial 3 years after radiotherapy and recovered afterwards, with telangiectasia being the most prevalent endoscopic finding. There was a statistically significant correlation between the VRS and clinical rectal bleeding. Cardiovascular disease, rectal wall V_65_, and VRS ≥ 3 in the first post-radiotherapy endoscopy were significantly associated with rectal bleeding and thus could be considered as risk factors.

## Data availability

The data that support the findings of this study are available upon reasonable requests to the corresponding author (W.P.; wonro.park@samsung.com).

### Supplementary Information

Supplementary Information.
