# Supplementary material for: Long-term findings of rectal endoscopy and rectal bleeding after moderately hypofractionated, intensity-modulated radiotherapy for prostate cancer
Source: Sci Rep. 2023 Dec 13;13:22099. doi: 10.1038/s41598-023-43202-x (PMC10719280; doi:10.1038/s41598-023-43202-x)
Supplement: Supplementary file 1 — Supplementary Information. [file 41598_2023_43202_MOESM1_ESM.docx]

**Supplementary Figure 1.** Flowchart of patient selection





**Supplementary Figure 2.** Cumulative incidence of rectal bleeding after radiotherapy, compared between the study cohort and the cohort before exclusion of patients without endoscopic findings (All patients). Incidences for all patients are shown as blue, orange, and red lines, and study cohort are shown as light blue, light orange, and light red lines.


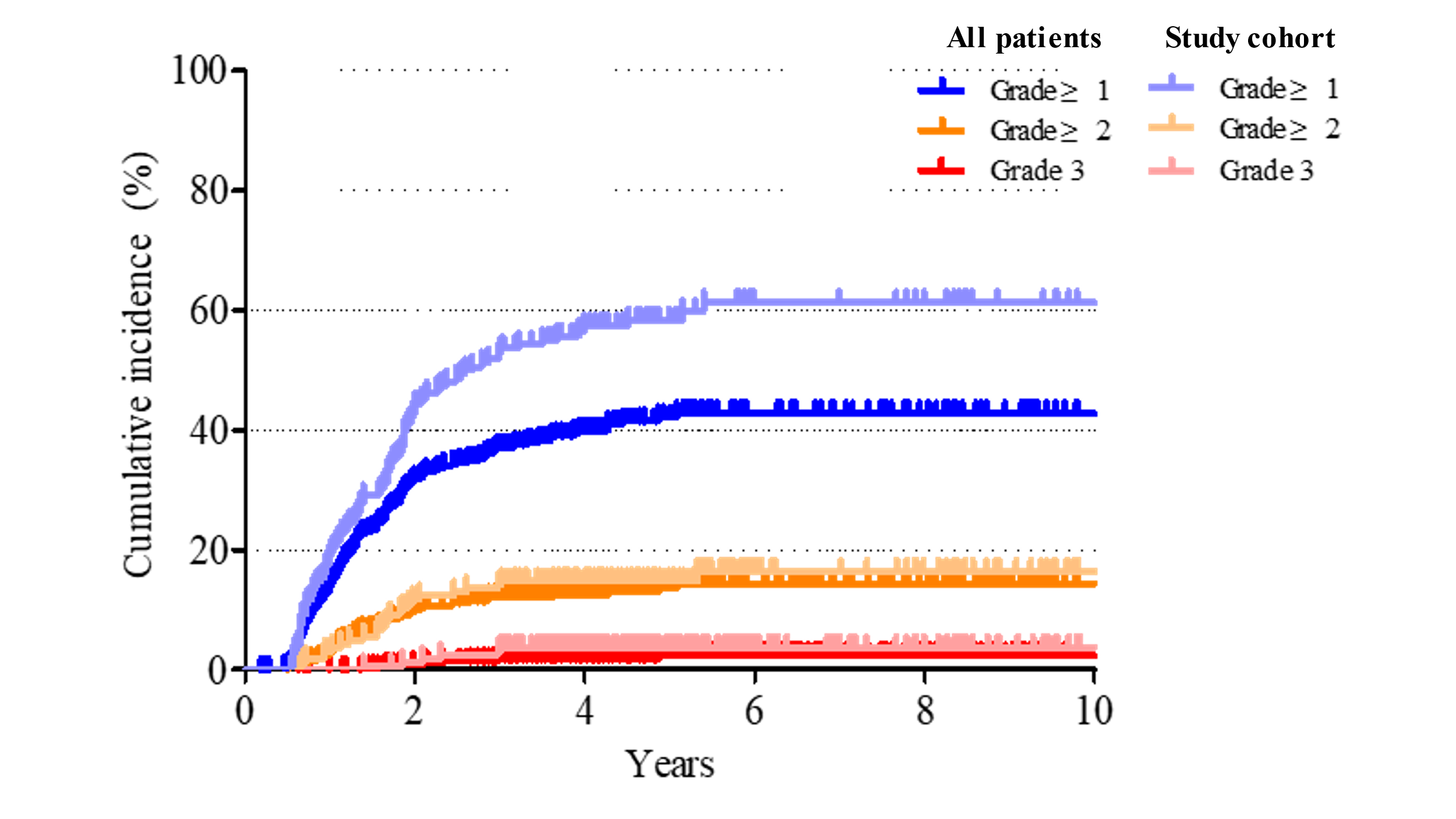


**Supplementary Table 1.** Changes in endoscopic findings over time.

|  | **Years after radiotherapy** | | | | |
| --- | --- | --- | --- | --- | --- |
|  | **0−1 (n = 149)** | **1−2 (n = 152)** | **2−3 (n = 129)** | **3−4 (n = 87)** | **4−5 (n = 53)** |
| VRS |  |  |  |  |  |
| 0 | 41 (27.5%) | 7 (4.6%) | 12 (9.3%) | 17 (19.5%) | 7 (13.2%) |
| 1 | 18 (12.1%) | 14 (9.2%) | 13 (10.1%) | 9 (10.3%) | 8 (15.1%) |
| 2 | 53 (35.6%) | 73 (48.0%) | 52 (40.3%) | 39 (44.8%) | 24 (45.3%) |
| 3 | 33 (22.1%) | 58 (38.2%) | 52 (40.3%) | 20 (23.0%) | 14 (26.4%) |
| 4 | 4 (2.7%) | 0 (0.0%) | 0 (0.0%) | 1 (1.1%) | 0 (0.0%) |
| 5 | 0 (0.0%) | 0 (0.0%) | 0 (0.0%) | 1 (1.1%) | 0 (0.0%) |
| Congested mucosa |  |  |  |  |  |
| Grade 0 | 69 (46.3%) | 84 (55.3%) | 71 (55.0%) | 47 (54.0%) | 34 (64.2%) |
| Grade 1 | 51 (34.2%) | 48 (31.6%) | 39 (30.2%) | 32 (36.8%) | 13 (24.5%) |
| Grade 2 | 26 (17.4%) | 19 (12.5%) | 19 (14.7%) | 7 (8.0%) | 6 (11.3%) |
| Grade 3 | 3 (2.0%) | 1 (0.7%) | 0 (0.0%) | 1 (1.1%) | 0 (0.0%) |
| Telangiectasia |  |  |  |  |  |
| Grade 0 | 47 (31.5%) | 9 (5.9%) | 14 (10.9%) | 21 (24.1%) | 8 (15.1%) |
| Grade 1 | 15 (10.1%) | 12 (7.9%) | 11 (8.5%) | 7 (8.0%) | 7 (13.2%) |
| Grade 2 | 59 (39.6%) | 74 (48.7%) | 52 (40.3%) | 39 (44.8%) | 24 (45.3%) |
| Grade 3 | 28 (18.8%) | 57 (37.5%) | 52 (40.3%) | 20 (23.0%) | 14 (26.4%) |
| Ulceration |  |  |  |  |  |
| Grade 0 | 132 (88.6%) | 149 (98.0%) | 129 (100.0%) | 85 (97.7%) | 53 (100.0%) |
| Grade 1 | 13 (8.7%) | 3 (2.0%) | 0 (0.0%) | 0 (0.0%) | 0 (0.0%) |
| Grade 2 | 4 (2.7%) | 0 (0.0%) | 0 (0.0%) | 1 (1.1%) | 0 (0.0%) |
| Grade 3 | 0 (0.0%) | 0 (0.0%) | 0 (0.0%) | 1 (1.1%) | 0 (0.0%) |

VRS, Vienna Rectoscopy Score.
